# Routine Repeat Head CT may not be Indicated in Patients on Anticoagulant/Antiplatelet Therapy Following Mild Traumatic Brain Injury

**DOI:** 10.5811/westjem.2014.10.19488

**Published:** 2014-12-01

**Authors:** Kevin C. McCammack, Charlotte Sadler, Yueyang Guo, Raja S. Ramaswamy, Nikdokht Farid

**Affiliations:** * University of California, San Diego, Department of Radiology, San Diego, California; † University of California, San Diego, Department of Emergency Medicine, San Diego, California; ‡ University of California, San Diego, School of Medicine, San Diego, California

## Abstract

**Introduction:**

Evaluation recommendations for patients on anticoagulant and antiplatelet (ACAP) therapy that present after mild traumatic brain injury (TBI) are controversial. At our institution, an initial noncontrast head computed tomography (HCT) is performed, with a subsequent HCT performed six hours later to exclude delayed intracranial hemorrhage (ICH). This study was performed to evaluate the yield and advisability of this approach.

**Methods:**

We performed a retrospective review of subjects undergoing evaluation for ICH after mild TBI in patients on ACAP therapy between January of 2012 and April of 2013. We assessed for the frequency of ICH on both the initial noncontrast HCT and on the routine six-hour follow-up HCT. Additionally, chart review was performed to evaluate the clinical implications of ICH, when present, and to interrogate whether pertinent clinical and laboratory data may predict the presence of ICH prior to imaging. We used multivariate generalized linear models to assess whether presenting Glasgow Coma Score (GCS), loss of consciousness (LOC), neurological or physical examination findings, international normalized ratio, prothrombin time, partial thromboplastin time, platelet count, or specific ACAP regimen predicted ICH.

**Results:**

144 patients satisfied inclusion criteria. Ten patients demonstrated initial HCT positive for ICH, with only one demonstrating delayed ICH on the six-hour follow-up HCT. This patient was discharged without any intervention required or functional impairment. Presenting GCS deviation (p<0.001), LOC (p=0.04), neurological examination findings (p<0.001), clopidogrel (p=0.003), aspirin (p=0.03) or combination regimen (p=0.004) use were more commonly seen in patients with ICH.

**Conclusion:**

Routine six-hour follow-up HCT is likely not indicated in patients on ACAP therapy, as our study suggests clinically significant delayed ICH does not occur. Additionally, presenting GCS deviation, LOC, neurological examination findings, clopidogrel, aspirin or combination regimen use may predict ICH, and, in the absence of these findings, HCT may potentially be forgone altogether.

## INTRODUCTION

Anticoagulation or antiplatelet (ACAP) therapy is frequently employed to treat or prevent vascular thromboembolic disease and associated complications.^1^,^2^ As a result, it is a relatively common occurrence for patients on ACAP regimens to present to emergency departments (ED) and trauma centers after traumatic brain injury (TBI), and recommendations for the appropriate evaluation of these patients are valuable. The imaging study of choice to evaluate for the presence of intracranial hemorrhage (ICH) is a non-contrast head computed tomography (HCT), which is able to detect the presence of acute intracranial hemorrhage with a rate of greater than 90%.^3^–^5^ However, review of the existing literature regarding its appropriate use in patients on ACAP therapy, particularly in cases of mild TBI, yields conflicting results with some authors concluding that imaging may not always be indicated,^6^,^7^ others contending that at least an initial HCT is prudent in patients on ACAP due to increased risk of injury without reliable pretest risk factors,^8^–^10^ and still others advocating serial imaging after an initial negative HCT to evaluate for delayed ICH.^11^

At our institution, patients on ACAP agents who present to the ED after mild TBI are routinely evaluated with an initial non-contrast HCT and a six-hour follow-up HCT if the first is negative to exclude delayed ICH prior to discharge. While this method is certainly reasonable given the lack of consensus data regarding this subject, the advisability of this or similar approaches has been called into question.^12^,^13^ In an era of stringent healthcare resource utilization measures and radiation safety concerns, expensive algorithms that rely heavily on imaging must be thoroughly evaluated. We sought to examine the current protocol at our institution for the evaluation of patients on ACAP agents presenting to the ED after mild TBI, including the overall yield of the routine six-hour follow-up HCT to exclude delayed ICH. We also aimed to assess whether or not clinical and laboratory data may be able to predict the presence or absence of ICH.

## METHODS

We performed a retrospective review of patients on ACAP therapy who sustained TBI and presented to our institution for subsequent evaluation from January 2012 through April 2013. Institutional review board exemption status was obtained, with informed patient consent waived. We queried our Radiology Information System for patient history information as provided by ordering clinicians when ordering a noncontrast HCT January 2012 through April 2013, with those patients noted to be on ACAP regimens selected for imaging and clinical review. Patients were then included in the study if they had suffered mild closed TBI, defined as having an initial Glasgow Coma Scale score (GCS) of 13–15, had completed both an initial noncontrast HCT as well as an interval follow-up HCT, and were on an ACAP agent or agents prior to the trauma. We included patients in the study even if they were on a single agent warfarin or dabigatran regimen with an initial international normalized ratio (INR) measurement in the normal range, commonly defined as less than 1.3, as the current practice in place at our institution makes no such distinction. Of note, all patients in this study who met the criteria for the six-hour follow-up HCT (i.e. presenting to the ED after mild TBI and on ACAP agents) underwent follow-up imaging, with no exceptions to this rule.

Patients were imaged on either a 64-detector row General Electric (GE) scanner with 0.625 mm detector width or a 320-detector row Toshiba scanner with 0.5 mm detector width. Imaging was performed from the skull base through the vertex, with multiple axial slice thickness reconstructions available in both bone and soft tissue algorithms. Coronal and sagittal reformats were universally available for interpretation. Patients were imaged at initial presentation and approximately six hours later, with some unavoidable variation in timing of the follow-up HCT due to demands of patient transport and scheduling.

A second-year radiology resident (KM) reviewed the final reports of all HCTs, with positive studies defined as having reported the presence of ICH, specifically epidural hematoma, subdural hematoma, subarachnoid hemorrhage, intraventricular hemorrhage, or parenchymal hemorrhage/contusion. Time interval between the two scans was also recorded. A board-certified neuroradiologist (NF) with over five years of experience reassessed equivocal cases when necessary.

The hospital electronic medical records system (EPIC) was examined with data logged in duplicate and independently by a second-year radiology resident (KM) and a fourth-year medical student (YG), recording the following using a standardized data abstraction form: age, sex, mechanism of injury, initial GCS, presence or absence of associated loss of consciousness (LOC), pertinent neurological and physical examination findings, specific ACAP regimen and treatment indication at the time of injury, INR, prothrombin time (PT), partial thromboplastin time (PTT), platelet count, any possible neurosurgical intervention, hospital encounter outcome, and any possible follow-up information available up to 30 days after the trauma. A senior emergency medicine resident (CS) blinded to the study hypotheses assessed the abstracted clinical data for accuracy, with any inadvertent discrepancies rectified by additional chart review.

Using multivariate generalized linear models, co-varying for the effects of age and sex throughout, we evaluated the relationship between intracranial hemorrhage as detected on either the initial or six-hour follow-up noncontrast HCT examination and the following: presenting GCS, presence or absence of associated loss of consciousness (LOC), presence or absence of neurological and pertinent physical examination findings, specific ACAP regimen (multivariate analysis assessing each agent effect individually), INR, PT, PTT, and platelet count. Statistical significance was assigned to p-values less than 0.05.

## RESULTS

One hundred forty-four patients (77 female, 67 male) satisfied the study inclusion criteria for the interrogated period of January 2012 through April 2013. The mean patient age was 74 years (median 77 years, range 25–96 years). ACAP medications in use at the time of TBI included warfarin, aspirin (ASA), clopidogrel, dipyridamole, dabigatran, or a combination of these agents (Table 1). Indications for ACAP therapy included atrial fibrillation, deep venous thrombosis/pulmonary thromboembolic disease, cardiac valve replacement, ischemic coronary artery disease/coronary artery stent, cerebral infarction or prior transient ischemic attack, New York Heart Association class III or greater congestive heart failure, hypercoagulable state, or a combination of these factors.

**Figure 1:**
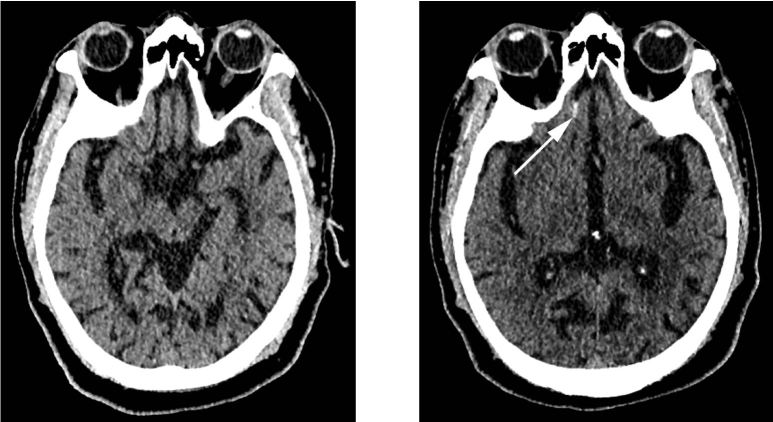
Noncontrast head computed tomography performed (top left) at initial presentation and (top right) six hours following injury demonstrates interval development of a small amount of subarachnoid hemorrhage in the right olfactory sulcus (arrow). This patient was on single-agent clopidogrel therapy and demonstrated no deviation from his functional baseline upon discharge.

Ten patients had an original presentation HCT positive for the presence of ICH (6.9%) while 134 were initially negative. Of the 134 patients with an initially negative HCT, only one was positive on the follow-up HCT (Figure 1), yielding a 0.7% incidence of delayed ICH. Of note, all 11 cases of ICH were deemed to most likely represent traumatic ICH given the constellation of radiographic findings and the absence of clinical findings to suggest hypertensive hemorrhagic infarcts. The single patient with delayed ICH had two follow-up HCT examinations over the next two days, which demonstrated stability followed by a slight decrease in size and conspicuity of the ICH, after which the patient was discharged at his baseline functional status with an outpatient follow-up appointment. No neurosurgical intervention was required and the patient had no readmission or post-traumatic sequelae greater than 30 days after the event.

**Figure 2:**
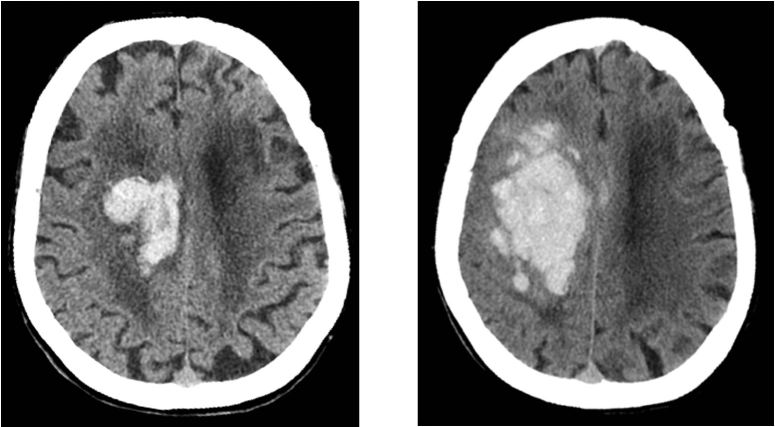
Noncontrast head computed tomography performed (top left) at initial presentation and (top right) six hours following injury demonstrates interval progression of the intraparenchymal and intraventricular hemorrhage centered predominately in the region of the right centrum semiovale, as well as corona radiata and body of the right lateral ventricle. This patient was on a coumadin and aspirin combination therapy with a presentation Glasgow Coma Scale of 13 and uneven pupillary response on neurological examination. This patient expired during the hospitalization.

Of the 10 patients with ICH on their initial HCT, six demonstrated stability of findings, three demonstrated interval worsening (Figure 2), and one actually improved on their subsequent examination. Two of the three patients who worsened between scans expired during the hospitalization secondary to their injuries. The third patient with interval worsening between scans was placed in a skilled nursing facility after discharge, with a significant, likely permanent, impairment from baseline. Two of the six patients with stable ICH on subsequent follow-up HCT were discharged to skilled rehabilitation facilities with mild persistent deficits, but were expected to return to their baseline. The remainder of patients with ICH on their initial HCT examinations were discharged home at their functional baseline without readmission or evidence of trauma-related symptoms greater than 30 days after the event.

Review of the electronic medical records of the 133 patients with initial and follow-up HCT both negative for ICH revealed no evidence of readmission or trauma-related symptoms for any patient at least 30 days after the event. As such, there is no available evidence that delayed ICH was missed in any of these patients.

We compared clinical and laboratory data available before imaging for patients with and without ICH on either the initial or follow-up HCT (Table 2). There was no significant difference between these groups in terms of age, sex, presence of physical examination findings reflecting trauma to the head, coagulation profile, or platelet count. Additionally, warfarin, dipyridamole, and dabigatran use did not significantly differ between the two groups. Those with ICH did demonstrate lower mean GCS at presentation than those without (14.3 versus 14.9, p<0.001) and LOC was more often seen in patients with ICH than those without (54.5% versus 26.5%, p<0.05). Pertinent neurological examination findings were present in 72.7% of patients with ICH compared to only 9.8% of patients without (p<0.001). ASA inclusion in therapeutic regimens was more common in patients with ICH (36.4% versus 13.6%, p<0.05) as was clopidogrel (36.4% versus 6.8%, p<0.01). Combination regimens were also more common in those with ICH (36.3% versus 15.9%, p<0.01). Please note, at our institution the ED and trauma departments are separate units which both treat this patient population, though these patients are treated identically. For the sake of brevity, we will refer to both of these units in this paper as the ED.

## DISCUSSION

Falls are the leading cause of trauma-related mortality for patients 65 years of age or older in the United States, with just under 8,000 deaths resulting from TBI in 2005.^14^ A 1% annual risk of spontaneous ICH is often quoted as resulting from anticoagulation therapy even without any antecedent trauma^15^, reasonably leading one to believe that TBI patients on ACAP agents must be treated with a high degree of clinical suspicion. It is critical to identify those patients on ACAP therapy with ICH early, as prompt coagulopathy reversal measures have been shown to reduce hemorrhagic progression and death.^16^ Studies evaluating the effects of ACAP agents on the development of ICH and resulting management recommendations are conflicting, with some suggesting specific criteria even for initial imaging and the most cautious advocating routine serial imaging to exclude delayed ICH in all patients on ACAP agents prior to discharge.^6^–^9^,^11^ Still other evidence suggests imaging of all elderly patients with mild TBI, even when there is no history of ACAP therapy.^17^

Our findings demonstrate that the use of a routine six-hour follow-up HCT in patients on ACAP treatment after mild TBI is of extremely low yield, with delayed ICH occurring in only one of 134 patients (0.7% incidence) in our study population. Furthermore, the one case of delayed ICH required no intervention and resulted in no sustained deviation from the patient’s baseline. This is in agreement with similarly structured retrospective studies that demonstrate a comparable incidence and a lack of clinical significance in those occurrences of delayed ICH.^12^,^13^ Tauber et al demonstrate a slightly higher incidence of delayed ICH and observed clinically significant consequences in half of those cases.^11^ It is interesting to note that their study included only patients on ASA therapy, which, in our study, proved to be an independent statistically significant risk factor for ICH when included in the therapeutic regimen, as did clopidogrel, while anticoagulant agents alone did not. It is possible that antiplatelet agents serve as a greater risk factor for ICH than anticoagulant medications, and that this accounts for or at least contributes to the discrepancy between our studies.

A prospective study by Nishijima et al. in fact addressed this question and compared the risk of immediate and delayed ICH in patients on warfarin versus those on clopidogrel, demonstrating an increased risk of immediate ICH in those patients on clopidogrel (12.0%) compared to those on warfarin (5.1%).^18^ This study largely agrees with our data, suggesting an increased risk of ICH in patients on antiplatelet agents over anticoagulant therapy, and their overall ICH rate of 7.0% is similar. Their rate of delayed ICH (0.4%) was also similar to ours; however, it was seen only in patients on warfarin. This may reflect the overall preponderance of patients on warfarin rather than clopidogrel within their study population, rather than indicating an increased risk of delayed ICH in patients on anticoagulant therapy versus antiplatelet agents. Regardless, the rate of delayed ICH is too low in both of our studies to draw a firm conclusion as to whether anticoagulant versus antiplatelet agents portend a larger comparative risk for delayed ICH. Overall, our data lends credence to this prior study, but also expands upon it by more comprehensively analyzing additional clinical factors that may predict the presence of ICH on HCT.

Combining the single patient who developed delayed ICH and the 10 patients with initially positive HCT examinations, the total incidence of ICH in our patient population was 7.6%. Gittleman et al. demonstrate a very similar rate of 7.8%, and they also show a statistically significant association between the absence of GCS abnormalities and neurological examination findings and negative initial HCT.^7^ Our findings support those conclusions. As demonstrated in Table 2, we observed statistically significant associations between decreased mean GCS at presentation, as well as neurological examination findings and the presence of ICH. Additionally, we demonstrate a statistically significant association between sustained LOC and the presence of ICH. As previously mentioned, the antiplatelet agents (ASA and clopidogrel) were statistically significant risk factors for the presence of ICH when included in therapeutic regimens, as were combination treatment strategies in general. Single agent warfarin, dabigatran, and dipyridamole regimens were not associated with statistically increased risk of ICH in this study. No patient with a normal GCS and neurological examination, without LOC at the time of the traumatic event, and on single anticoagulant agent therapy demonstrated ICH at any point during this study.

This study questions the advisability of routine six-hour follow-up HCT after an initial negative HCT for the exclusion of ICH in patients on ACAP agents prior to discharge, suggesting that clinically significant delayed ICH does not occur in these patients. Previous studies suggest that clinical findings herald the progression of ICH when present, and that routine follow-up HCT is not necessary, even in patients with known ICH.^19^,^20^ Additionally, while routine serial imaging even in patients with proven ICH is shown not to influence clinical outcomes, it needlessly increases hospital length of stay.^21^ In an era where cost containment and radiation safety are particularly emphasized, and acute care facility throughput is critical, it may therefore be most prudent to abandon such an approach, instead observing these patients clinically and performing additional imaging only if clinical examination changes dictate such prior to discharge.

## LIMITATIONS

Our study is limited primarily by the relatively low sample numbers and retrospective design. While we were able to achieve significance with our statistical analyses, a greater positive ICH sample size may provide additional information, particularly if there are any factors at play that may predict the evolution of ICH, when present, between serial scans. This can be better assessed using a prospective design in the future. Additionally, we were limited in our ability to obtain clinical information, besides what had been recorded in the electronic records, due to the retrospective design. While we have no evidence of any missed cases of delayed ICH, it may be possible that some patients did develop eventual symptoms after discharge and presented to a different institution for evaluation and treatment. A prospective design would allow for the establishment of a specific follow-up protocol to more thoroughly assess for this possibility.

## CONCLUSION

Despite limitations, our study provides an important data point in the ongoing debate regarding the appropriate management of patients on ACAP therapy who sustain mild TBI. Based on these results, for patients with normal presentation GCS and neurological examinations, no associated LOC, on single anticoagulant therapy and no antiplatelet agent, we may be able to forego initial imaging altogether. Furthermore, a six-hour follow-up HCT is of extremely low yield in general and is likely not indicated, as our data suggests that clinically significant delayed ICH does not occur.

## Figures and Tables

**Table 1:** Demographic information of patient population in a study of patients on anticoagulant and antiplatelet therapy after mild traumatic brain injury.*

	# of patients
Mechanism of injury	
Mechanical fall	107
Syncope	17
Intoxicated/found down	8
Motor vehicle accident	6
Assault	4
Seizure activity	2
Agents in use at time of trauma	
Warfarin	134
Aspirin	22
Clopidogrel	13
Dabigatran	2
Dipyridamole	1
Combination regimen	25

* Age (mean in years [range]) of patient population was 74 (25–96).

**Table 2:** Clinical and laboratory data compared between those patients with negative initial and follow-up head computed tomography and those positive for intracranial hemorrhage on either the initial or follow-up examination.

	Negative	Positive	P	Odds ratio	95% CI
Age	73.8	79.9	0.19		
Sex			0.91		
Male (%)	47.0	45.5		0.95	0.28–3.28
Female (%)	53.0	54.5		1.42	0.41–4.87
GCS*	14.9	14.3	<0.001		
LOC (%)*	26.5	54.5	0.04	9.44	2.57–34.73
Neuro exam (%)*	9.8	72.7	<0.001	24.62	5.80–104.42
Physical exam (%)	58.3	54.5	0.64	0.87	0.25–3.00
Warfarin (%)	93.9	90.9	0.61	0.64	0.07–5.64
Clopidogrel (%)*	6.8	36.4	0.003	11.17	2.33–53.59
Aspirin (%)*	13.6	36.4	0.03	4.74	1.16–19.36
Dipyridamole (%)	0.8	0	0.68		
Dabigatran (%)	1.5	0	0.65		
Combination(%)*	15.9	36.3	0.004	4.11	1.59–10.82
INR	2.4	2.8	0.51		
PT	29.3	31.7	0.75		
PTT	39.5	37.9	0.55		
Platelet Count	200.7	218.9	0.35		

*GCS,* Glasgow Coma Scale score; *LOC,* loss of consciousness; *INR,* international normalized ratio; *PT,* prothrombin time; *PTT,* partial thromboplastin time

* Denotes statistical significance.

## References

[1] Kirley K, Qato DM, Kornfield R (2012). C. National trends in oral anticoagulant use in the United States, 2007 to 2011. Circ Cardiovasc Qual Outcomes.

[2] McLaughlin GE (2002). Aspirin for the primary prevention of cardiovascular events. Ann Intern Med.

[3] Byyny RL, Mower WR, Shum N (2008). Sensitivity of noncontrast cranial computed tomography for the emergency department diagnosis of subarachnoid hemorrhage. Ann Emerg Med.

[4] Morgenstern LB, Luna-Gonzales H, Huber JC (1998). Worst headache and subarachnoid hemorrhage: prospective, modern computed tomography and spinal fluid analysis. Ann Emerg Med.

[5] Sidman R, Connolly E, Lemke T (1996). Subarachnoid hemorrhage diagnosis: lumbar puncture is still needed when the computed tomography scan is normal. Acad Emerg Med.

[6] Garra G, Nashed A, Capobianco L (1999). Minor head trauma in anticoagulated patients. Acad Emerg Med.

[7] Gittleman AM, Ortiz AO, Keating DP (2005). Indications for CT in patients receiving anticoagulation after head trauma. AJNR Am J Neuroradiol.

[8] Li J, Brown J, Levine M (2001). Mild head injury, anticoagulants, and risk of intracranial injury. Lancet.

[9] Reynolds FD, Dietz PA, Higgins D (2003). Time to deterioration of the elderly, anticoagulated, minor head injury patient who presents without evidence of neurologic abnormality. J Trauma.

[10] Brewer ES, Reznikov B, Liberman RF (2011). Incidence and predictors of intracranial hemorrhage after minor head trauma in patients taking anticoagulant and antiplatelet medication. J Trauma.

[11] Tauber M, Koller H, Moroder P (2009). Secondary intracranial hemorrhage after mild head injury in patients with low-dose acetylsalicylate acid prophylaxis. J Trauma.

[12] Peck KA, Sise CB, Shackford SR (2011). Delayed intracranial hemorrhage after blunt trauma: are patients on preinjury anticoagulants and prescription antiplatelet agents at risk?. J Trauma.

[13] Kaen A, Jimenez-Roldan L, Arrese I (2010). The value of sequential computed tomography scanning in anticoagulated patients suffering from minor head injury. J Trauma.

[14] Thomas KE, Stevens JA, Sarmiento K (2008). Fall-related traumatic brain injury deaths and hospitalizations among older adults--United States, 2005. J Safety Res.

[15] Hart RG, Boop BS, Anderson DC (1995). Oral anticoagulants and intracranial hemorrhage. Facts and hypotheses. Stroke.

[16] Ivascu FA, Howells GA, Junn FS (2005). Rapid warfarin reversal in anticoagulated patients with traumatic intracranial hemorrhage reduces hemorrhage progression and mortality. J Trauma.

[17] Mack LR, Chan SB, Silva JC (2003). The use of head computed tomography in elderly patients sustaining minor head trauma. J Emerg Med.

[18] Nishijima DK, Offerman SR, Ballard DW (2012). Immediate and delayed traumatic intracranial hemorrhage in patients with head trauma and preinjury warfarin or clopidogrel use. Ann Emerg Med.

[19] Sifri ZC, Homnick AT, Vaynman A (2006). A prospective evaluation of the value of repeat cranial computed tomography in patients with minimal head injury and an intracranial bleed. J Trauma.

[20] Velmahos GC, Gervasini A, Petrovick L (2006). Routine repeat head CT for minimal head injury is unnecessary. J Trauma.

[21] Nayak NV, Medina B, Patel K (2013). Neurologic outcome of minimal head injury patients managed with or without a routine repeat head computed tomography. J Trauma Acute Care Surg.

